# Biomechanical correlation at the knee joint between static lunge and single-leg drop landing - a comparative study among three different toe directions

**DOI:** 10.1186/s40634-019-0208-2

**Published:** 2019-10-30

**Authors:** Kengo Harato, Aiko Sakurai, Yutaro Morishige, Shu Kobayashi, Yasuo Niki, Takeo Nagura

**Affiliations:** 1 Department of Orthopedic Surgery, Keio University School of Medicine, 35 Shinanomachi, Shinjuku-ku, Tokyo, 160-8582 Japan; 2 Institute for Integrated Sports Medicine, Keio University School of Medicine, 35 Shinanomachi, Shinjuku-ku, Tokyo, 160-8582 Japan

## Abstract

**Background:**

Toe direction is a critical factor affecting knee biomechanics during various movements including closed kinetic chain (CKC) exercise and landing tasks. Physiotherapists always concentrate on the control of toe direction during CKC exercise as a first step for athletes, as it is believed that correction of toe direction during CKC exercise is important to maintain the appropriate toe position even in high demanding activities such as landing tasks. The purpose of the present study was to investigate knee biomechanics during CKC exercise as well as landing tasks with three different toe directions, and clarify whether biomechanical parameters during CKC exercise would be related to those during landing tasks.

**Methods:**

A total of 23 male recreational level athletes (mean age = 20.0 ± 1.1 yrs) participated. Tegner activity score were 7 for all male subjects. First, the subjects performed weight-bearing static lunge tests (SL) under three different toe directions, including 0 degrees (Toe-neutral: TN), 20 degrees (Toe-In: TI), and - 20 degrees (Toe-out: TO). Thereafter, SLDL was done under three different toe directions. Three-dimensional knee kinematics and kinetics at 60 degrees of knee flexion were calculated. As a statistical analysis, Pearson's correlation coefficient was used to evaluate the relationship between SL and SLDL. The statistical significance level wasset at *P*=0.05.

**Results and Conclusions:**

Knee abduction angle showed significant correlation between SL and SLDL in all three different directions (TI: r=0.631, *p*<0.001, TN: r=0.678, *p*<0.001, TO: r=0.572, *p*<0.001). In terms of knee internal rotation, strong correlation was also found (TI: r=0.846, *p*<0.001, TN: r=0.791, *p*<0.001, TO: r=0.749, *p*<0.001). In addition, external knee abduction moment presented significant correlation in all three different directions (TI: r=0.574, *p*<0.001, TN: r=0.499, *p*<0.01, TO: r=0.469, *p*<0.01). From the present study, significant correlation between SL and SLDL was found in knee abduction angle, knee internal rotation, and external knee abduction moment under all three different directions including TI, TN, and TO. Physiotherapist should take care of toe direction and reform the movements especially for athletes who present malalignment of the knee joint during SL with TI or TO to prevent ACL injury in landing tasks.

## Introduction

Generally, closed kinetic chain (CKC) exercise such as the static lunge (SL) is useful for rehabilitation to avoid overloading of anterior cruciate ligament (ACL) especially in the early postoperative period after the surgery of ACL reconstruction, as CKC exercise induces co-contraction of the agonist and antagonist muscles (Cho et al. 2013; Escamilla et al. 1998; Keays et al. 2013; Lin et al. 2007; Romero-Franco et al. 2017). Besides, neuromuscular training should be added to strength training. Thereafter, patients with ACL reconstruction can start with the next phase only if specific goals of the previous phase are achieved. Jumping tasks are used as the next step of the postoperative rehabilitation after SL exercise. Clinically, physiotherapists must pay attention to a quality of movement for prevention of re-injuries. Qualitative criteria, including dynamic knee valgus, knee flexion angle, hip and trunk control, can play an important role in postoperative rehabilitation and prevention of second ACL injury (Ucar et al. 2014; van Melick et al. 2016). For example, the occurrence of dynamic knee valgus when landing from a jump increases the risk of primary ACL injury as well as second ACL injury.

According to previous studies, foot position during SL and jumping tasks would be related to malalignment of the knee joint. Tran et al. investigated the effect of foot landing position on biomechanical risk factors associated with ACL injury using healthy participants during double-leg drop landing, and indicated that toe-in landing position exacerbated biomechanical risk factors associated with ACL injury, while toe-out landing position decreased these factors (Tran et al. 2016). In addition, Teng et al. evaluated single-leg drop landing (SLDL) for eleven male recreational basketball players under three different toe directions and concluded that they should avoid extreme toe-out foot rotation positions to minimize undesirable knee valgus loading associated with non-contact ACL injury risks (Teng et al. 2017). Besides, Ishida et al. assessed the knee rotation during dynamic knee valgus and evaluated whether the knee rotation should be affected by toe direction using healthy females during SL (Ishida et al. 2014). They concluded that the knee rotated externally during dynamic knee valgus, and the knee rotation was affected by toe direction. Therefore, toe direction is a critical factor affecting knee biomechanics during various movements including CKC exercise and landing tasks. Physiotherapists always concentrate on the control of toe direction during CKC exercise as a first step for athletes, as it is believed that correction of toe direction during CKC exercise is important to maintain the appropriate toe position even in high demanding activities such as landing tasks. However, little attention has been paid to the biomechanical relationship between SL and SLDL.

The purpose of the present study was to investigate knee biomechanics during SL as well as SLDL with three different toe directions, and clarify whether biomechanical parameters during SL would be related to those during SLDL or not. It was hypothesized that knee kinematics and kinetics during SL could be correlated with those during SLDL.

## Methods

### Subjects

A total of 23 male recreational level athletes (mean age = 20.0 ± 1.1 yrs., mean height = 172.9 ± 6.0 cm, mean weight = 64.3 ± 7.2 kg) participated in the present study. None of the subjects had any history of major injuries to the trunk and lower extremities. Thirteen subjects joined in college soccer team, and ten subjects joined in college ski club activity. Tegner activity level was 7 for all males. An informed consent form approved by the institutional review board at our university (#20080054) was signed by each subject.

### Procedures

**Fig. 1:**
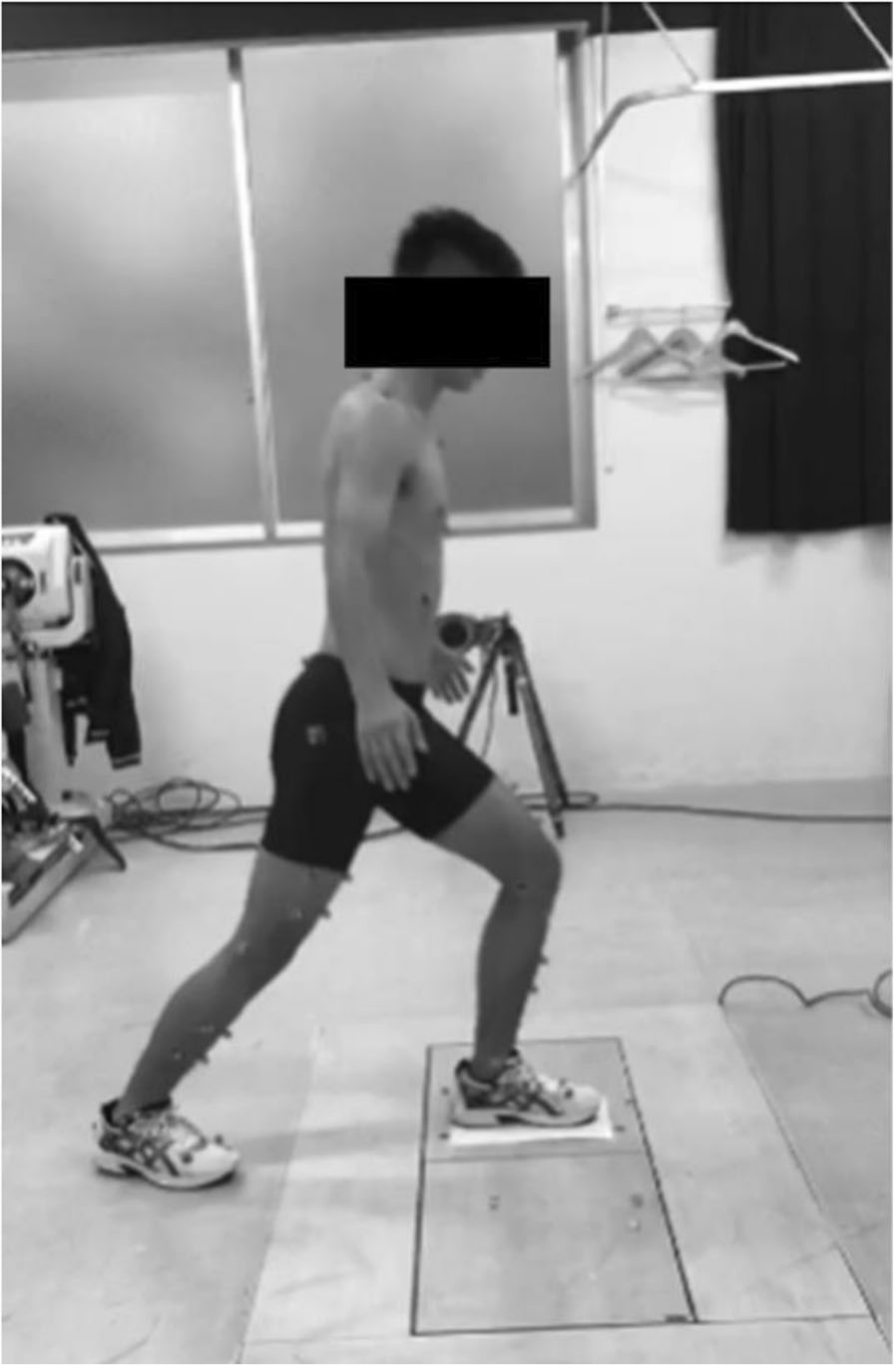
Static Lunge was done for the non-dominant limb. The measured foot was placed in front and the unmeasured foot was placed behind the measured foot. Subject was asked to tilt the trunk by 30 degrees and the knee flexion on measured side was set at 60 degrees

**Fig. 2:**
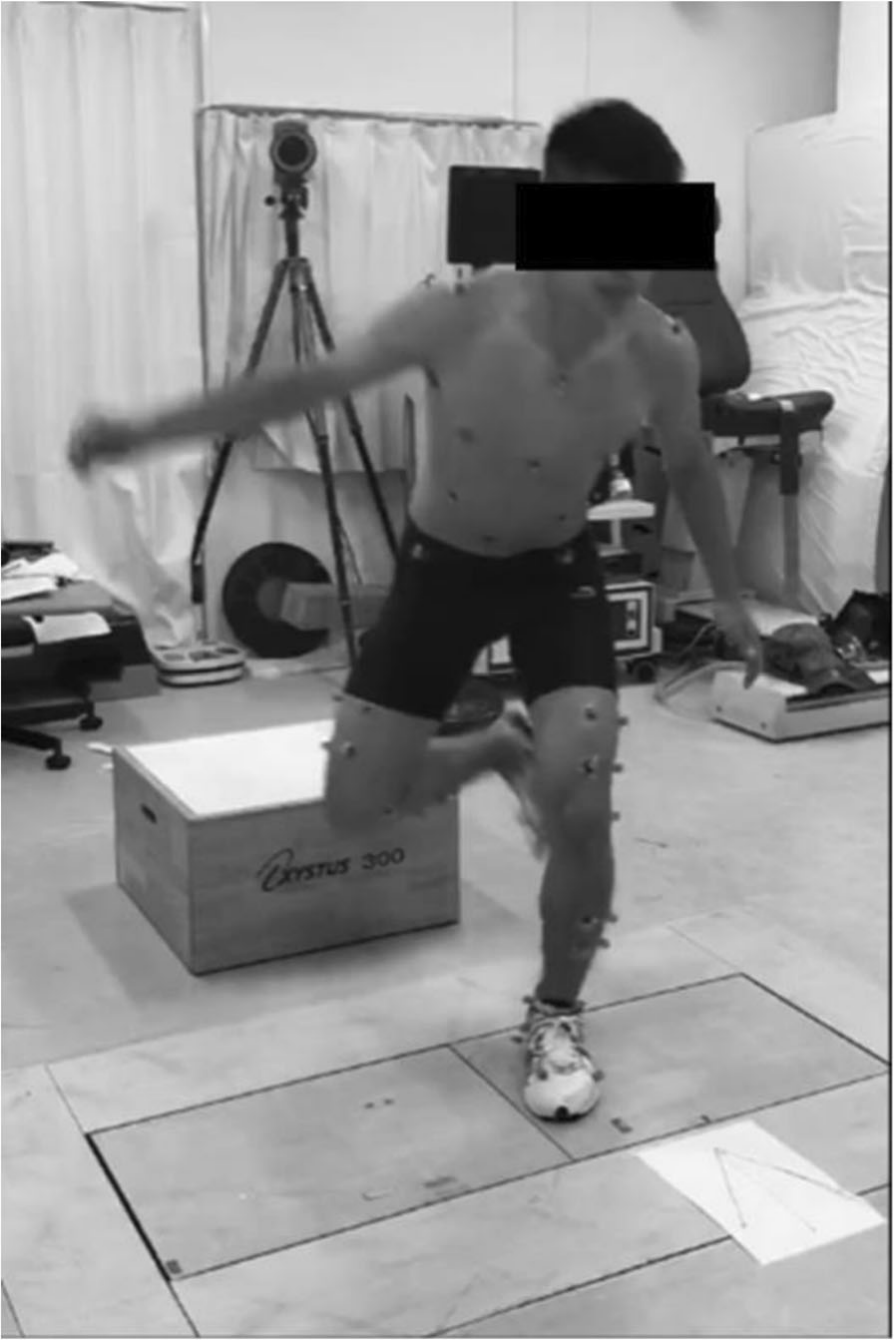
Single-leg drop landing was performed for the non-dominant limb. Single-leg drop landing tasks were jumping from a 30-cm high box to a distance of 25% of their height away from the box, down to force plates

The subjects performed static lunge (SL) and thereafter single-leg drop landing (SLDL). The subjects performed weight-bearing SL on measured limb under three different toe directions, including 0 degrees (Toe Neutral: TN), 20 degrees (Toe-In: TI), and − 20 degrees (Toe-out: TO) (Fig. 1). Toe angles were set using the same paper sheet for each subject. To perform SL, the measured foot was placed in front and the contralateral foot was placed behind the measured foot. Subject was asked to tilt the trunk by 30 degrees and the knee flexion on measured side was set at 60 degrees. Angles of trunk tilt and knee flexion were confirmed using goniometer for each athlete. The same model of sport shoes (Maximizer 20, Mizuno Corporation, Tokyo, Japan) was provided to all participants to reduce the influence of footwear based on each participant’s foot size. SLDL tasks were jumping from a 30-cm high box to a distance of 25% of their height away from the box, down to force plates without any specific instructions about the arm position. Similarly, SLDL was done under three different toe directions, including TN, TI, and TO (Fig. 2). Toe angles were also set using the same paper sheet for each subject. The non-dominant leg (23 left) was chosen for the measurement. The dominant leg was defined as the leg with which each athlete preferred to kick a ball. After performing SL and SLDL 5 to 6 times including warm-ups, two successful trials were recorded for each subject using a motion analysis system. Motion analysis system was consisted of 8 cameras (120 frames/s; Oqus, Qualisys, Sweden), two force plates (frequency 600 Hz; AM6110, Bertec, Columbus, OH, USA), and 46 retro-reflective markers (14 mm in diameter). Successful trials were determined by the achievement of clear contact within force plates to the force plate without marker drop. An anatomical model was created by digitizing the following standard bony landmarks: bilateral acromion processes, xiphoid process, suprasternal notch, 7th cervical vertebra, 10th thoracic vertebra, bilateral anterior and posterior superior iliac spines, bilateral iliac crests, bilateral greater trochanters, bilateral lateral and medial femoral epicondyles, bilateral lateral and medial malleoli, bilateral posterior heels, bilateral medial cuneiforms, bilateral great toes, and bilateral heads of the 5th metatarsals. Four additional tracking markers were placed on each of the frontal aspects of the thigh and shank. Some markers (bilateral medial epicondyles and medial malleoli) were removed after calibration, and only tracking markers were left on the participants throughout all data collection. These anatomical markers provided a reference marker set for construction of a skeletal model using a commercial biomechanical analysis software program (C-motion Company, Rockville, MD, USA) (Whatman et al. 2011, 2013). All markers in the present study were positioned by an experienced musculoskeletal physiotherapist (AS). All anatomical markers were attached directly to the skin with double sided adhesive tape. The motion of markers was recorded by Qualisys Track Manager Software (version 2.7). Three-dimensional knee kinematics (°) and kinetics (Nm/kg) at 60 degrees of knee flexion were calculated using Visual 3D. Knee internal rotation was defined as tibial rotation with respect to the femur. In this software program, the static trial was used to create geometric objects and to represent each body segment. All lower limb segments were modeled as frusta of cones. Together these segments formed a 6-degree-of-freedom, rigid link biomechanical model. Joints in the model are defined as places where the distal end of one segment meets the proximal end of another segment (Whatman et al. 2011, 2013).

### Statistical analysis

A power analysis was performed using G*Power (v3.1.9.2, Heinrich-Heine University, Düsseldorf, Germany). Using a large effect size of 0.5 for a one-way repeated measures Analysis of Variance, a sample size of 23 was required (β = .80, α = .05). As a statistical analysis, Pearson’s correlation coefficient was used to evaluate the relationship between SL and SLDL. The statistical significance level was set at *P* = 0.05. All statistical analyses were performed using SPSS® for Windows version 23 software (Microsoft, Chicago, IL, USA).

## Results

### Kinematics

**Fig. 3:**
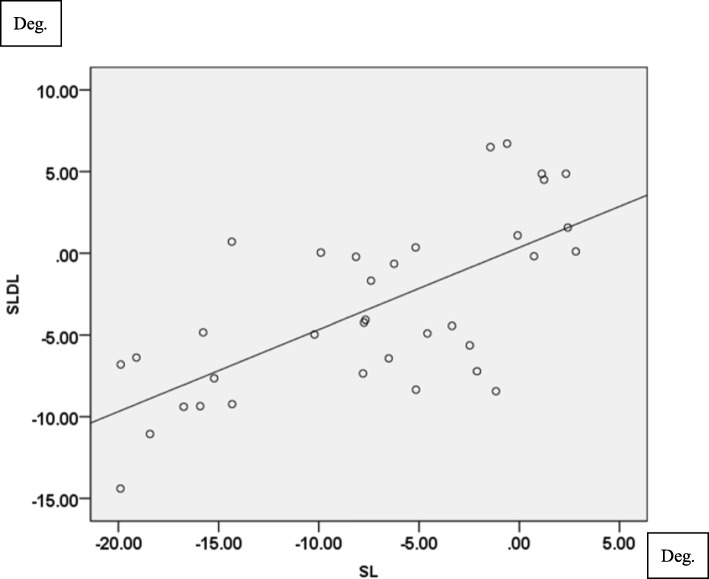
Correlation of knee abduction angle (°) between SL and SLDL in TN. (*r* = 0.678, *P*<0.001)

**Fig. 4:**
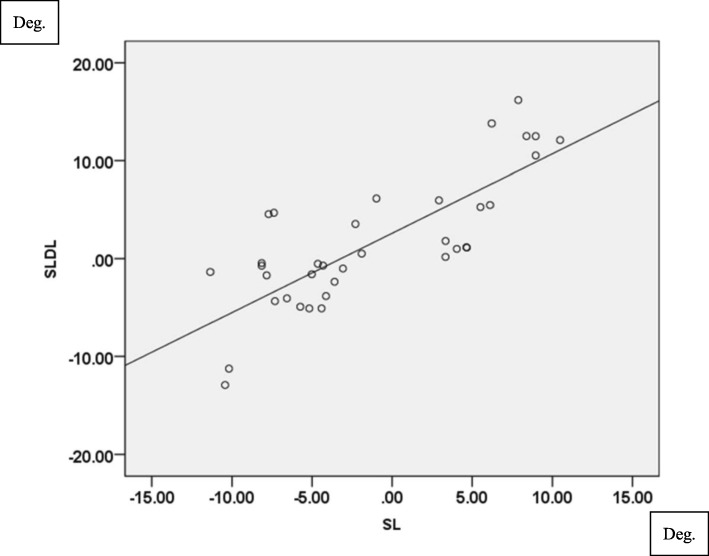
Correlation of tibial internal rotation angle (°) between SL and SLDL in TN. (*r* = 0.791, *P*<0.001)

Knee abduction angle showed significant correlation between SL and SLDL in all three different directions (TI: *r* = 0.631, *p* < 0.001, TN: *r* = 0.678, *p* < 0.001 in Fig. 3, TO: *r* = 0.572, *p* < 0.001). In terms of knee internal rotation, strong correlation was found in all three different directions (TI: *r* = 0.846, *p* < 0.001, TN: *r* = 0.791, *p* < 0.001 in Fig. 4, TO: *r* = 0.749, *p* < 0.001).

### Kinetics

**Fig. 5:**
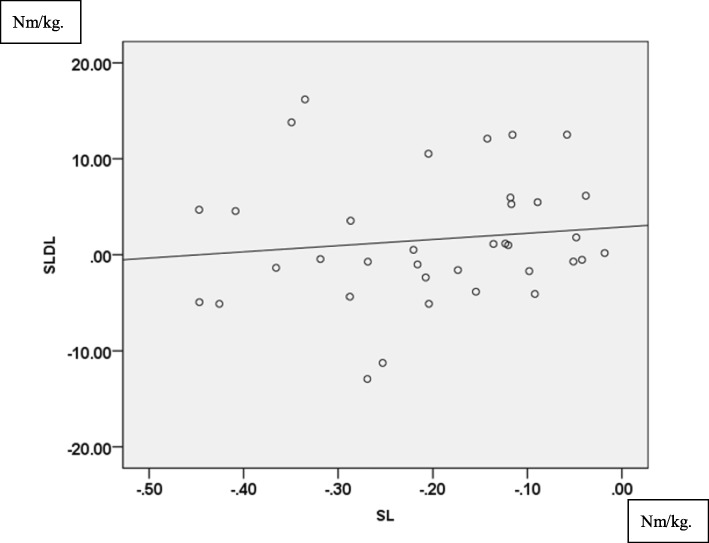
Correlation of external knee abduction moment (Nm/kg) between SL and SLDL in TN. (*r* = 0.499, *P*<0.01)

As to knee kinetics, external knee flexion moment did not have correlation between these movements (TI: *r* = − 0.11, *p* = 0.529, TN: *r* = − 0.007, *p* = 0.969, TO: *r* = − 0.022, *p* = 0.900). On the other hand, external knee abduction moment presented significant correlation in all three different directions (TI: *r* = 0.574, *p* < 0.001, TN: *r* = 0.499, *p* < 0.01 in Fig. 5, TO: *r* = 0.469, *p* < 0.01). Regarding knee internal rotation moment, no significant correlation was found (TI: *r* = 0.313, *p* = 0.067, TN: *r* = 0.263, *p* = 0.122, TO: *r* = 0.269, *p* = 0.118). Even if knee abduction angle, tibial internal rotation angle, and external knee abduction moment had significant correlations, values were notably greater in SLDL than in SL especially for external knee abduction moment, as SLDL was a higher demanding activity compared to SL.

## Discussion

The result of the present study partly supported the hypothesis that knee kinematics and kinetics during SL could be correlated with those during SLDL. The most important finding of the current investigation was that significant correlation between SL and SLDL was found in knee abduction angle, knee internal rotation, and external knee abduction moment under all three different directions including TI, TN, and TO, though absolute value was different between these movements.

The occurrence of dynamic knee valgus when landing from a jump increases the risk of ACL injury. According to the previous studies, valgus loading would be a contributing factor in ACL injury mechanism and tibial internal tibial rotation was coupled with valgus motion (Koga et al. 2010). Hewett et al. reported that female athletes with increased knee abduction angle at initial contact, peak knee abduction angle, and peak knee abduction moment would be correlated with an increased risk of non-contact ACL injury during drop vertical jump (Hewett et al. 2005, 2015). Moreover, Kimura et al. evaluated 17 right-handed female college badminton players using a 3-dimensional motion analysis system, and suggested that increased knee valgus angle and moment following back-stepping to the backhand-side might be related to the higher incidence of ACL injury during single-leg landing after overhead stroke (Kimura et al. 2012). Therefore, knee abduction angle, external knee abduction moment and internal tibial rotation angle were extremely important for the assessment of non-contact ACL injury. In the present study, significant correlations between SL and SLDL were found in knee abduction angle, knee internal rotation, and external knee abduction moment under three different toe directions. Based on the previous studies, toe-in and toe-out might lead to the malalignment of the knee joint (Sakurai et al. 2019). It is helpful for physiotherapists to take caution of the toe-neutral position for the patients with ACL injury during SL exercise, as it is believed that correction of toe direction during SL is important to maintain the appropriate toe position even in high demanding activities such as SLDL.

Several limitations should be noted in the present study. First, all subjects in the present study were male athletes. Results of female athletes remain unknown. Although females have a greater risk of ACL injury than males, males were selected in the present study as all examiners for the present study were males in our laboratory. Second, stiffness of the ankle was not evaluated, even if the toe direction was confirmed using the same sheets. According to the previous study, restrictions in passive ankle dorsiflexion range of motion might contribute to a landing with less flexion at the knee joint (Dowling et al. 2018). Third, muscular activation using surface electromyography did not be assessed. Fourth, skin motion error was not assessed, and thus it was possible that the accuracy during SLDL was not equal to that during SL. Lastly, the present study was evaluated only at 60 degrees of knee flexion, while ACL injuries typically occur near extension during landing tasks. However, the results of the current study provide valuable information when considering the biomechanical correlation between SL and SLDL for appropriate rehabilitation.

## Conclusions

From the present study, significant correlation between SL and SLDL was found in knee abduction angle, knee internal rotation, and external knee abduction moment under all three different directions including TI, TN, and TO. In addition, as TI and TO may lead to the malalignment of the knee joint based on previous studies, physiotherapist should take care of toe direction and reform the movements especially for athletes who present malalignment of the knee joint during SL with TI or TO to prevent ACL injury in landing tasks.

## Data Availability

All supporting data can be provided based on request to the authors.

## Abbreviations

ACL: Anterior cruciate ligament
CKC: Closed kinetic chain
SL: Static lunge
SLDL: Single-leg drop landing
TI: Toe-in
TN: Toe- neutral
TO: Toe-out
